# pERK, pAKT and p53 as tissue biomarkers in erlotinib-treated patients with advanced pancreatic cancer: a translational subgroup analysis from AIO-PK0104

**DOI:** 10.1186/1471-2407-14-624

**Published:** 2014-08-28

**Authors:** Steffen Ormanns, Jens T Siveke, Volker Heinemann, Michael Haas, Bence Sipos, Anna Melissa Schlitter, Irene Esposito, Andreas Jung, Rüdiger P Laubender, Stephan Kruger, Ursula Vehling-Kaiser, Cornelia Winkelmann, Ludwig Fischer von Weikersthal, Michael R Clemens, Thomas C Gauler, Angela Märten, Michael Geissler, Tim F Greten, Thomas Kirchner, Stefan Boeck

**Affiliations:** Institute of Pathology, Ludwig-Maximilians-University of Munich, München, Germany; Department of Internal Medicine II, Klinikum Rechts der Isar, Technische Universität München, München, Germany; Department of Internal Medicine III and Comprehensive Cancer Center, Klinikum Grosshadern, Ludwig-Maximilians-University of Munich, München, Germany; German Cancer Consortium (DKTK), Heidelberg, Germany; German Cancer Research Center (DKFZ), Heidelberg, Germany; Institute of Pathology, University of Tübingen, Tübingen, Germany; Institute of Pathology, Technische Universität München, München, Germany; Institute of Medical Informatics, Biometry and Epidemiology, Ludwig-Maximilians-University of Munich, München, Germany; Practice for Medical Oncology, Landshut, Germany; Department of Internal Medicine, Krankenhaus Lutherstadt-Wittenberg, Lutherstadt-Wittenberg, Germany; Department of Oncology, Gesundheitszentrum St. Marien GmbH, Amberg, Germany; Department of Internal Medicine I, Klinikum Mutterhaus Trier, Trier, Germany; Department of Medicine (Cancer Research), West German Cancer Center, University of Duisburg-Essen, Essen, Germany; Department of Surgery, University of Heidelberg, Heidelberg, Germany; Department of Gastroenterology and Oncology, Klinikum Esslingen, Esslingen am Neckar, Germany; Department of Gastroenterology, Hepatology and Endocrinology, Medical School Hannover, Hannover, Germany

**Keywords:** Biomarker, EGFR, Erlotinib, Pancreatic cancer

## Abstract

**Background:**

The role of pERK, pAKT and p53 as biomarkers in patients with advanced pancreatic cancer has not yet been defined.

**Methods:**

Within the phase III study AIO-PK0104 281 patients with advanced pancreatic cancer received an erlotinib-based 1^st^-line regimen. Archival tissue from 153 patients was available for central immunohistochemistry staining for pERK, pAKT and p53. Within a subgroup analysis, biomarker data were correlated with efficacy endpoints and skin rash using a Cox regression model.

**Results:**

Fifty-five out of 153 patients were classified as pERK^low^ and 98 patients as pERK^high^; median overall survival (OS) was 6.2 months and 5.7 months, respectively (HR 1.29, p = 0.16). When analysing pERK as continuous variable, the pERK score was significantly associated with OS (HR 1.06, 95% CI 1.0-1.12, p = 0.05). Twenty-one of 35 patients were pAKT^low^ and 14/35 pAKT^high^ with a corresponding median OS of 6.4 months and 6.8 months, respectively (HR 1.03, p = 0.93). Four out of 50 patients had a complete loss of p53 expression, 20 patients a regular expression and 26 patients had tumors with p53 overexpression. The p53 status had no impact on OS (p = 0.91); however, a significant improvement in progression-free survival (PFS) (6.0 *vs* 1.8 months, HR 0.24, p = 0.02) and a higher rate of skin rash (84% *vs* 25%, p = 0.02) was observed for patients with a regular p53 expression compared to patients with a complete loss of p53.

**Conclusion:**

pERK expression may have an impact on OS in erlotinib-treated patients with advanced pancreatic cancer; p53 should be further investigated for its potential role as a predictive marker for PFS and skin rash.

**Trial registration:**

NCT00440167 (registration date: February 22, 2007).

**Electronic supplementary material:**

The online version of this article (doi:10.1186/1471-2407-14-624) contains supplementary material, which is available to authorized users.

## Background

Despite significant efforts in clinical research in pancreatic cancer during the last decade, only moderate progress has been achieved. Main steps forward were generated with the introduction of adjuvant chemotherapy as a standard of care after curative-intent resection (yielding in a potential long-term survival or even cure in about 20% of patients resected) and more recently, with the introduction of novel combination chemotherapy regimens (e.g. FOLFIRINOX and gemcitabine/nab-paclitaxel) for the treatment of metastatic disease [1, 2]. However, several large phase III trials investigating targeted agents (in unselected patient populations) have failed with only erlotinib (in addition to standard gemcitabine) achieving a moderate gain in overall survival (OS) [3, 4].

To date, no validated tissue biomarker is available that would allow a prognostic patient stratification or even the prediction of treatment efficacy in pancreatic cancer. Several molecular predictive markers like the human equilibrative nucleoside transporter 1 (hENT1; for the efficacy of gemcitabine) and secreted protein acidic and rich in cysteine (SPARC; for nab-paclitaxel) are currently under investigation with in part inconclusive results up to now [3, 5, 6]. Regarding the use of EGFR targeting agents in advanced pancreatic cancer, the tumor *KRAS* status may play a role as a prognostic or even predictive biomarker: based on previous translational results from AIO-PK0104, a large randomized phase III trial comparing a treatment sequence of gemcitabine + erlotinib followed by capecitabine *vs* capecitabine + erlotinib followed by gemcitabine in patients with advanced disease, patients with a *KRAS* wildtype may have a prolonged survival compared to patients with *KRAS* exon 2 mutations [7]. Other groups have confirmed these data, and the work of Kim and colleagues even suggested that *KRAS* may serve as a predictive biomarker for the efficacy of erlotinib [8, 9]. Based on previous analyses conducted on archival formalin fixed paraffin embedded (FFPE) tissue from AIO-PK014 no other marker besides *KRAS* showed a correlation with survival endpoints or objective response: data on EGFR protein expression, *EGFR* gene amplification, PTEN expression and on *EGFR* intron 1 polymorphism did not show - despite previous pre-clinical evidence - a correlation with efficacy study endpoints [10–12].

We thus decided to additionally analyze downstream targets of the EGFR pathway, namely phospho-ERK (pERK) and phospho-AKT (pAKT) as potential biomarkers in advanced pancreatic cancer. Both represent effector molecules in the EGFR downstream signalling network, pERK for the RAS/RAF/MEK cascade and pAKT is involved in the PI3K/mTOR pathway [13, 14]. Previous evidence (mainly derived from surgical series and/or in non-erlotinib treated patients) suggests a potential role of pERK and pAKT in pancreatic cancer biology and both markers additionally may represent a more appropriate way to assess EGFR pathway activation compared to single upstream markers like EGFR itself [14–17]. As increasing pre-clinical evidence from mouse models and also from genome-wide analyses revealed an important role of p53 in the pathogenesis of pancreatic cancer the investigators decided to also include this marker in the current translational analysis from AIO-PK0104 [18, 19]. This decision specifically was also based on recent data showing a potential interaction of p53 with *KRAS* signalling [19].

The aim of this retrospective translational biomarker study based on the prospective AIO-PK0104 trial thus was to determine the frequency of alterations in pERK, pAKT and p53 in erlotinib treated patients with advanced pancreatic cancer, as well as to investigate if there is a correlation of biomarker data with efficacy (progression-free survival, PFS and OS) and safety endpoints (skin rash) from the clinical data set.

## Methods

### Translational patient population

Adult patients (18 to 75 years) with a histologically or cytologically confirmed diagnosis of treatment-naïve, advanced exocrine pancreatic cancer (stage III and IV) were eligible for AIO-PK0104. Overall, 281 patients were randomized and 274 patients were eligible for the intention-to-treat (ITT) population; detailed results from the clinical study have been published in 2013 [7]. Archival FFPE tissue, which was obtained during routine pre-therapeutic diagnostic procedures, was requested retrospectively from the participating centers/pathologists for the translational study. Cytological specimens were not included. FFPE histological tissue was accepted independent of its origin, e.g. surgical or biopsy specimens from primary pancreatic tumor, lymph nodes or distant metastases. The study had approval of the local ethical committees in all participating German centers (see Additional file 1: Table S1) and patients gave written informed consent prior to any study-specific procedure. This study was conducted according to GCP/ICH guidelines and according to the Declaration of Helsinki and was registered at ClinicalTrials.gov, number NCT00440167.

### Analyses of molecular tissue biomarkers

The current translational analyses from AIO-PK0104 were performed at the Ludwig-Maximilians-University of Munich by SO and TK (pERK), at the Technical University of Munich by JTS, AMS and IE (pAKT and p53/*TP53*) as well as at the University of Tübingen by BS (p53). All available FFPE tumor blocks were checked for quality, tissue integrity and tumor content (HE staining) by a pathologist (SO) in a blinded manner. All involved pathologists that analyzed tumor tissue were blinded to the clinical study results.

#### Immunohistochemistry

Immunohistochemistry (IHC) was performed on a Ventana Benchmark XT autostainer using the XT UltraView diaminobenzidine kit (Ventana Medical Systems, Oro Valley, AZ, USA). The primary antibodies were as follows: monoclonal anti-pERK1/2 antibody (rabbit anti-phospho-p44/42 MAPK [Thr202/Tyr204] clone 20G11, Cell Signaling Technology, Danvers, MA, USA), anti-pAKT (Santa Cruz, sc-135650, 1:30, heat mediated antigen retrieval) and anti-p53 (Novocastra, DO-7, 1:200, heat mediated antigen retrieval).

#### pERK expression

For the examination of pERK IHC a scoring system analogously to a score developed for PTEN by Loupakis *et al.* was applied [20]: the nuclear and cytoplasmic staining intensity (0 - no, 1 - weak, 2 - moderate, and 3 - strong staining) were added to the score for the percentage of positive cells (0 - negative, 1 - less than 25%, 2 - 25% to 50%, 3 - more than 50% positive staining cells) and finally nuclear and cytoplasmic score were summarized (score 0–12; see Additional file 2: Figure S1). Specimens were defined as pERK^high^ if the total score was 6 or higher.

#### pAKT expression

Cytoplasmatic and nuclear pAKT expression by IHC in tumor cells was scored analogously to the pERK score described above. If the score was 5 or higher, samples were defined as pAKT^high^. Cases that exhibited no staining in stromal or inflammatory cells (as internal controls) were omitted (see Additional file 3: Figure S2, D-F).

#### p53 expression

p53 expression has been evaluated as completely lost (no reaction in tumor cells but positive internal control), variable (no to strong) nuclear expression as normal and diffusely strong as aberrant overexpression (see Additional file 3: Figure S2, A-C).

#### *TP53*mutational analysis

The investigators additionally selected 12 good- and poor-risk study patients based on clinical data for objective response and time-to-treatment failure for 1^st^-line therapy (TTF1, a pre-defined study endpoint [7]) in order to analyze the *TP53* mutation status (exon 5 to 8) by Sanger sequencing. The following primers were used: Ex-5 F:ATC TGT TCA CTT GTG CCC TG, Ex-5R:AAC CAG CCC TGT CGT CTC TC, Ex-6 F.AGG GTC CCC AGG CCT CTG AT, Ex-6R:CAC CCT TAA CCC CTC CTC CC, Ex-7 F:CCA AGG CGC ACT GGC CTC ATC, Ex-7R:CAG AGG CTG GGG CAC AGC AGG, Ex-8 F.TTC CTT ACT GCC TCT TGC TT and Ex-8R:TGT CCT GCT TGC TTA CCT CG.

### Statistical analyses

All statistical analyses for the translational study of the AIO-PK0104 trial were performed centrally at the Ludwig-Maximilians-University of Munich, Institute of Medical Informatics, Biometry and Epidemiology by RPL. Translational biomarker data were correlated with efficacy (PFS and OS) and safety study endpoints (skin rash) using univariate analyses. As appropriate, biomarker results were handled as dichotomous/categorical variables (e.g. pAKT^low^*vs* pAKT^high^, p53 expression) or as continuous variables (e.g. linear scoring system 0 to 12 for pERK). Time-to-event endpoints were analyzed with the Kaplan-Meier method; differences were compared using the log-rank test with a 2-sided p-value of ≤0.05 being regarded as statistically significant.

## Results

### Patient characteristics

For the current analysis, FFPE tumor blocks were initially available from 153 of the 281 randomized patients. The ITT study population consisted of 274 eligible patients, and 153 patients were selected for the current translational patient population; detailed patient characteristics are summarized in Table 1. With regard to important baseline parameters (e.g. age, gender, stage of disease, performance status), no significant imbalances between the ITT population and the translational study population were apparent.

**Table 1:** **Baseline patient characteristics: Intention-to-treat population (n = 274) and translational study population (n = 153)**

Parameter	Intention-to-treat				Translational			
	*Gem + E (n = 143)*		*Cap + E (n = 131)*		*Gem + E (n = 85)*		*Cap + E (n=68)*	
	No.	%	No.	%	No.	%	No.	%
Age [years]								
*Median*	65		63		63		64	
*Range*	32 - 78		38 - 75		32 - 75		42 - 75	
Gender								
*Male*	82	57	83	63	49	58	41	60
*Female*	61	43	48	37	36	42	27	40
Stage of disease								
*Locally advanced*	21	15	22	17	13	15	13	19
*Metastatic*	122	85	109	83	72	85	55	81
Performance status								
*KPS 60-80%*	50	35	49	37	29	34	30	44
*KPS 90-100%*	85	59	79	60	50	59	38	56
*Missing*	8	6	3	2	6	7	0	0
Previous surgery	8	6	17	13	7	8	8	12
Weight loss during three								
months before								
randomisation [kg]								
*Median*	5		7		5.5		7	
*Range*	0 - 47		0 - 45		0 - 20		0 - 35	
Baseline CA 19–9 [U/ml]^1^								
*Median*	1999		1756		2632		1255	
*Range*	1 - 700000		1 - 1000000		2 - 700000		1 - 1000000	

Abbreviations: *Cap* Capecitabine, *E* Erlotinib, *Gem* Gemcitabine, *KPS* Karnofsky performance status; ^1^n = 245 / 274.

### Frequency of alterations in pERK, pAKT and p53 and their correlation with efficacy endpoints

Within Table 2, each of the 3 analysed markers was categorized as a dichotomous variable and a correlation between selected baseline patient characteristics and molecular marker results was performed. No significant differences were obvious for the biomarker results based on clinical characteristics; of note, although the numbers were small, all 4 patients with a p53 loss had a good pre-treatment performance status and 2 of them (50%) had locally advanced disease (whereas in patients with regular p53 expression only 5% had locally advanced pancreatic cancer).

**Table 2:** **Selected patient characteristics and molecular marker results in the translational study population (n = 153)**

Parameter	p-ERK (n = 153)		p-AKT (n = 35)		p53 (n = 50)		
	*pERK* ^*low*^	*pERK* ^*high*^	*pAKT* ^*low*^	*pAKT* ^*high*^	*Loss*	*Regular*	*Overexpression*
	*n = 55*	*n = 98*	*n = 21*	*n = 14*	*n = 4*	*n = 20*	*n = 26*
	n	n	n	n	n	n	n
Age [years]							
*Median*	66	63 p = 0.45	64	61 p = 0.27	61	61	65 p = 0.50
Gender							
*Male*	32	58 p = 1.00	12	7 p = 0.74	1	10	15 p = 0.48
*Female*	23	40	9	7	3	10	11
Stage of disease							
*Locally advanced*	7	19 p = 0.37	1	1 p = 1.00	2	1	3 p = 0.08
*Metastatic*	48	79	20	13	2	19	23
Performance status							
*KPS 60-80%*	23	36 p = 0.73	8	3 p = 0.46	0	4	12 p = 0.07
*KPS 90-100%*	31	57	13	11	4	16	14
CA 19–9 [U/ml]							
*Median*	1118	2030 p = 0.19	1975	3154 p = 0.74	1894	3084	1539 p = 0.95
Treatment arm							
*Gem + E*	27	58 p = 0.24	9	10 p = 0.17	2	12	14 p = 0.91
*Cap + E*	28	40	12	4	2	8	12

Abbreviations: *Cap* Capecitabine, *E* Erlotinib, *Gem* Gemcitabine, *KPS* Karnofsky performance status.

#### pERK

**Figure 1:**
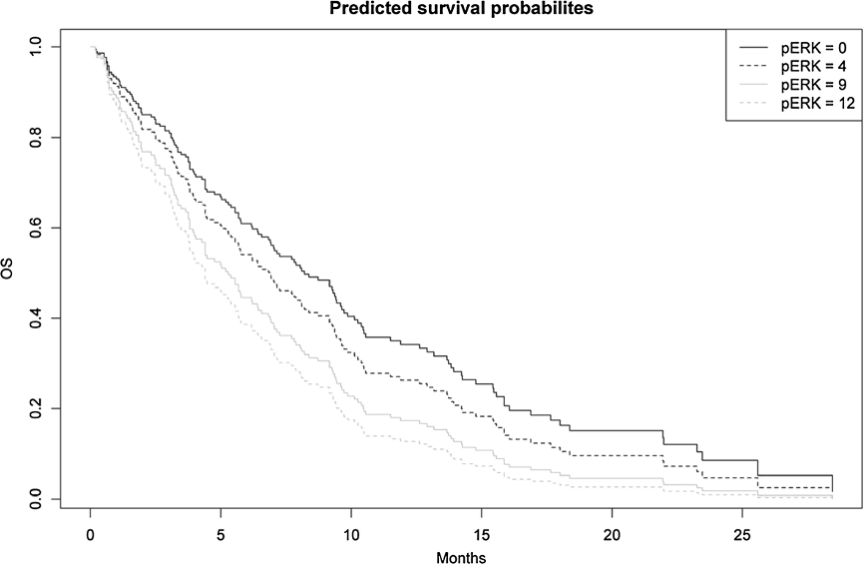
**Predicted overall survival (OS) probabilities (derived from a Cox model) based on the 4 exemplary pERK IHC score levels 0, 4, 9 and 12 (n = 153; p[** ***log rank*** **] = 0.050).**

pERK IHC staining was performed on 153 samples, classifying 55 patients as pERK^low^ (36%) and 98 patients as pERK^high^ (64%), with a median score level of 7 (range 0 to 12). pERK expression showed no correlation with *KRAS* status (p = 0.32), EGFR protein expression (p = 0.38) or *EGFR* gene amplification (p = 1.00), respectively. The pERK status had no impact on objective treatment response (p = 0.91). Median OS for patients classified as pERK^low^ was 6.2 months compared to 5.7 months in the pERK^high^ group (HR 1.29, 95% CI 0.90-1.83; p = 0.16) (Table 3). When analysing pERK as a continuous variable, an increase in the hazard for death by a factor of 1.06 was detected for each level of the pERK expression score of 0 to 12 (HR 1.06, 95% CI 1.0-1.12; p[*log rank*] = 0.050, p[*likelihood ratio*] = 0.047). Within Figure 1, these data are illustrated graphically by showing the predicted survival probabilities (derived from a Cox model) based on 4 exemplary pERK IHC score levels.

**Table 3:** **Correlation of biomarker results (** ***dichotomous*** **variables) with efficacy parameters: Progression-free survival (PFS) and overall survival (OS)**

*Biomarker*	*n*	*Median PFS*			*Median OS*		
*(Alteration)*		Mo.	HR (95% CI)	p	Mo.	HR (95% CI)	p
p-ERK	153						
*pERK* ^*low*^	55	3.1	1.01 (0.69-1.47)	0.98	6.2	1.29 (0.90-1.83)	0.16
*pERK* ^*high*^	98	3.2			5.7		
p-AKT	35						
*pAKT* ^*low*^	21	2.5	1.04 (0.46-2.34)	0.92	6.4	1.03 (0.51-2.11)	0.93
*pAKT* ^*high*^	14	3.9			6.8		
p53	50						
*Loss*	4	1.8			8.1		
*Regular*	20	6.0	0.24 (0.08-0.79)		7.0	1.10 (0.37-3.25)	
*Overexpression*	26	2.5	0.47 (0.16-1.40)	0.03^+^	5.0	1.22 (0.42-3.57)	0.91^+^

Abbreviations: *CI* Confidence interval, *HR* Hazard ratio, *Mo* Months.

(^+^global p value).

#### pAKT

After analysing pERK and p53, samples with an adequate amount of tumor tissue (for a usable pAKT IHC scoring) were available from 35 patients only. Of those, 21 (60%) were classified as pAKT^low^ and 14 (40%) as pAKT^high^; the pAKT IHC score level in the analyzed subgroup ranged from 2 to 10. As shown in Table 3, the pAKT status had no significant impact on PFS or OS. Due to the lower numbers, no analysis as a continuous variable was performed for pAKT.

#### p53 immunohistochemistry and *TP53*mutational analyses

**Figure 2:**
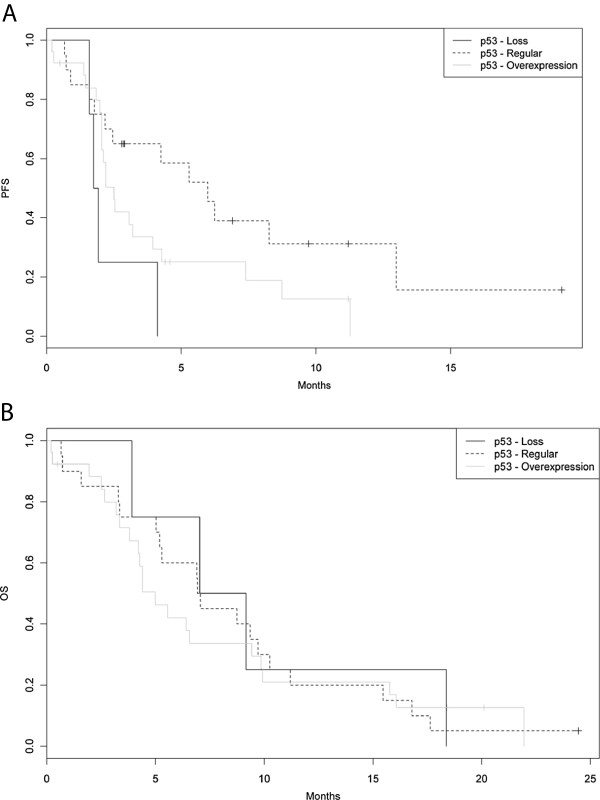
**Correlation of efficacy endpoints with the p53 IHC expression level. A)** Progression-free survival (PFS, n = 50; p[global] = 0.03). **B)** Overall survival (OS, n = 50; p[global] = 0.91).

A p53 status assessment by IHC could be carried out successfully on 50 tumor samples: 4 patients (8%) had a complete loss of p53 expression, 20 patients (40%) a regular p53 expression and 26 patients (52%) a p53 overexpression, respectively. In 43 analysable patients, p53 expression did not correlate with the *KRAS* mutation status (wildtype *vs* mutation; p = 0.32). Also no significant association between p53 and the objective response rate to 1^st^-line treatment was obvious (p = 0.26). The p53 expression had no significant impact on OS (8.1 *vs* 7.0 *vs* 5.0 months, p[*global*] = 0.91; Table 3 and Figure 2B); patients with an intact p53 expression had a significantly prolonged PFS (6.0 months) compared to patients with a loss of p53 (1.8 months) or p53 overexpression (2.5 months; p[*global*] = 0.03) (see Table 3 and Figure 2A).

We furthermore used archival DNA samples for performing an additional post-hoc explorative analysis on *TP53* gene mutations: for this post-hoc investigation, we selected 12 good- and poor-risk patients and performed *TP53* sequencing of exons 5 to 8. Detailed results of these analyses are summarized within Table 4: we detected 6 missense mutations in 5 tumors, with one additional silent mutation (R248R). All patients with a prolonged disease control (TTF1 ≥ 10 months) had *TP53* wildtype tumors, with 1 tumor (case no. 33) carrying the silent *TP53* mutation R248R. However, we also found patients that had a rapid disease progression (e.g. case no. 98) that was classified as *TP53* wildtype. One patient (case no. 99) had 2 mutations in exon 5 of p53 (T155I & V137M) and was clinically characterized by a rapid disease progression with a TTF1 of 0.5 months only (Table 4).

**Table 4:** ***TP53*** **mutation analysis [exon 5–8] in 12 selected good- and poor-risk study patients from AIO-PK0104 (in correlation with KRAS status and p53 IHC)**

*Case*	Exon 5	Exon 6	Exon 7	Exon 8	*p53 expression by IHC*	*KRAS status (Exon 2)*	*Best response by imaging (RECIST)*	*TTF1 [Mo.]*
198	wt	wt	wt	wt	*regular*	*wt*	*PR*	*19.1*
8	n.a.	n.a.	R249S	n.a.	*n.a.*	*mut*	*SD*	*6.0*
197	n.a.	wt	n.a.	wt	*Regular*	*mut*	*PD*	*1.8*
60	n.a.	wt	n.a.	wt	*n.a.*	*mut*	*PR*	*6.3*
89	wt	wt	wt	wt	*n.a.*	*wt*	*SD*	*9.8*
64	wt	wt	wt	wt	*n.a.*	*mut*	*SD*	*10.0*
50	n.a.	D208N	wt	wt	*n.a.*	*mut*	*PD*	*2.0*
33	n.a.	wt	R248R*	wt	*n.a.*	*mut*	*SD*	*19.6*
134	V147I	wt	wt	wt	*Overexpression*	*mut*	*SD*	*3.9*
132	n.a.	wt	wt	V274I	*Overexpression*	*mut*	*PD*	*2.2*
99	T155I &	wt	wt	wt	*n.a.*	*mut*	*PD*	*0.5*
	V137M							
98	wt	wt	wt	wt	*n.a.*	*wt*	*PD*	*1.7*

Abbreviations: *IHC* Immunohistochemistry, *Mo* Months, *mut* Mutation, *PD* Progressive disease, *PR* Partial response, *SD* Stable disease, *TTF1* Time-to-treatment failure after 1^st^-line therapy, *wt* Wildtype.

(*silent mutation).

### Correlation of biomarkers results with skin rash

Neither the pERK nor the pAKT status correlated with the occurrence of skin rash (any grade I-III): the corresponding data for each marker (analyzed as dichotomous variable) are summarized in Table 5. Patients with a loss of p53 in their tumor had a rash incidence during treatment with erlotinib of only 25%, whereas patients with a regular p53 expression had a rate for skin rash of 84% (p[*global*] = 0.04).

**Table 5:** **Correlation of biomarkers results with the occurrence of skin rash (any grades, I-III)**

*Biomarker*	*n*	*Skin rash [%]*		p
		No rash	Any rash (grade I-III)	
p-ERK	153			
*pERK* ^*low*^	55	29	71	0.41
*pAKT* ^*high*^	98	36	64	
p-AKT	35			
*pERK* ^*low*^	21	33	67	0.49
*pAKT* ^*high*^	14	46	54	
p53	50			
*Loss*	4	75	25	0.04^+^
*Regular*	20	16	84	
*Overexpression*	26	39	61	

(^+^gobal p value).

## Discussion

Based on translational data from controlled prospective clinical trials no prognostic or predictive tissue biomarkers have yet been defined in advanced pancreatic cancer. Some large phase III studies investigating anti-EGFR treatment strategies like erlotinib or cetuximab (in comparison to a single-agent gemcitabine control arm) were - at least up to now - not able to define prognostic or predictive biomarkers [21, 22]. Within the current translational subgroup analysis of AIO-PK0104 we thus tried to exploratively analyze if the EGFR pathway ‘downstream’ mediators pERK and pAKT could serve as biomarkers in advanced pancreatic cancer. Previous evidence from mainly small surgical series indicated that both pERK and pAKT may have a biological role in pancreatic cancer, however with in part inconclusive results on their prognostic role [15–17]. Regarding pERK, 64% of the 153 analyzed tumor samples from AIO-PK0104 were classified as pERK^high^; a significant impact on OS was found when the pERK expression score (0 to 12) was analyzed as a continuous variable (Figure 1). The observed HR of 1.06 (95% CI 1.0-1.12) reflects an increase in the hazard for death by a factor of 1.06 for each level of the pERK expression score of 0 to 12. If this association is a prognostic or a predictive phenomenon remains unclear, as all patients treated within AIO-PK0104 received an erlotinib-containing 1^st^-line regimen [7]. Nevertheless, based on the fact that the pERK status had no impact on PFS or response, one might hypothesize that the observed effect on OS is rather prognostic than predictive for the efficacy of erlotinib. In contrast, the IHC expression pattern of pAKT could successfully be determined in a smaller subgroup of only 35 patients and showed no impact on survival endpoints.

The potentially most interesting and hypothesis-generating findings from the current translational analysis of AIO-PK0104 are derived from the data on p53. The role of the transcription factor p53 as a tumor suppressor in several human cancers is well known; however its role as a prognostic or predictive biomarker remains largely unclear to date [23]. A loss of p53 is thought to stimulate tumor growth and dissemination, whereas a regular p53 expression might be associated with a more favorable disease biology. The biological role of a p53 overexpression in human tumors still is not understood entirely. Currently it also remains a matter of debate if p53 should be assessed by IHC or by mutational analysis; most authors indeed recommend combining IHC and gene-sequencing as the most reliable prognostic tool [23]. Of the 50 samples from our study cohort assessed for p53 by IHC, 4 showed a complete loss, 20 a regular expression and 26 an overexpression. Interestingly, a loss of p53 and p53 overexpression were associated with a dismal PFS, but on the other hand had no impact on OS (Table 3 and Figure 2). Thus one might conclude (as postulated by other authors earlier) that the overexpression status of p53 might lead to a functional loss of p53 resulting in the same biological effects like the absence of p53 expression by IHC. When we looked at the association of p53 with the occurrence of skin rash (a well known side-effect of erlotinib and other anti-EGFR drugs and a predictive ‘clinical marker’ for drug efficacy) we found rash - of any grade - in 84% of the patients whose tumor carried a regular p53 expression, whereas only 25% of patients with a p53 loss developed rash during treatment with erlotinib (p = 0.02). Thus one might hypothesize a potential role for p53 as a biomarker for rash and/or as a predictive marker for the efficacy of erlotinib.

Based on the interesting p53 findings from the IHC analyses, we additionally performed Sanger sequencing of the *TP53* gene (exon 5 to 8) in 12 selected good- and poor-risk AIO-PK0104 study patients (see Table 4). Simultaneous p53 IHC data were unfortunately available in 4 out of 12 patients only: two patients with *TP53* wildtype had a regular p53 expression, and 2 patients with a *TP53* mutation had a p53 overexpression. All patients with a prolonged disease control (TTF1 ≥ 10 months) had *TP53* wildtype tumors, including one patient whose tumor carried the silent *TP53* mutation R248R. These data of course should be regarded pre-liminary, but they provide early evidence of a potential important role for the p53/*TP53* status as a biomarker in patients treated with anti-EGFR agents. This evidence is supported by recent data presented at the 2013 European Cancer Congress on neoadjuvant cetuximab-containing chemoradiotherapy (CRT) in locally advanced rectal cancer: translational data from a subgroup of patients (n = 144) treated within the EXPERT-C trial showed that the *TP53* mutation status might serve as an independent predictive biomarker for cetuximab efficacy [24]. In detail, for *TP53* wildtype patients (48%) the addition of cetuximab to CRT was associated with a statistically significant advantage in PFS (HR 0.23, p = 0.02) and OS (HR 0.16, p = 0.02). In multivariate analyses, this interaction remained significant even after adjusting for other prognostic factors and *KRAS* status. In contrast, for *TP53* mutant patients (52%) no improvement in survival endpoints was observed for the addition of cetuximab to neoadjuvant CRT [24].

The main limitation from our current retrospective translational study arises from the fact that only a subgroup of the study patients could be analyzed for the biomarkers pERK, pAKT and p53. This circumstance mainly is based on the fact that tumor samples were limited in our study collective (specifically after already having performed detailed molecular analyses on *KRAS*, EGFR and PTEN [10]), a persisting and well known hurdle for many international collaborative groups that conduct translational research in advanced pancreatic cancer [25]. In light of the small sample size, specifically the data on pAKT and p53 should be regarded carefully and hypothesis-generating only and they clearly need prospective validation in a larger cohort of patients. Additionally one should also keep in mind that the assessment of an IHC-based scoring system may have several methodological limitations, specifically when analyzing categorical data by using predefined cut-offs (that often themselves have no clear biological rationale). We tried to overcome some of these limitations by analyzing our pERK data as continuous variable (see Figure 1); an approach that possibly better reflects the biological function of a molecular marker compared to results obtained when dichotomizing biomarker data (e.g. high *vs* low or positive *vs* negative, respectively).

The investigators are aware of the fact that the clinical value of adding erlotinib to standard gemcitabine is limited in an unselected patient population with metastatic pancreatic cancer. This specifically holds true in the context of recent phase III data on FOLFIRINOX and nab-paclitaxel, that both provide a meaningful and relevant advance in the treatment of pancreatic adenocarcinoma [26, 27]. Nevertheless, there is a subgroup of patients that derives a clinically relevant benefit of adding erlotinib to gemcitabine, namely those patients that develop skin rash during treatment. In these patients, median survival times of about 10 months can be expected - a time frame that is in the same range of the FOLFIRINOX or gemcitabine/nab-paclitaxel data [4, 7, 26, 27]. Up to now, we unfortunately are not able to molecularly define this scientifically interesting subgroup and the authors thus believe that pancreatic cancer researchers should continue their efforts to define molecular subgroups that might derive benefit from EGFR-targeting agents in this harmful disease. In this context, some novel hypothesis-generating findings could be obtained by the current translational analysis of AIO-PK0104; however, as these results will currently have no direct impact on clinical practice, a prospective scientific validation of our pre-liminary findings is recommended.

## Conclusions

For this translational study downstream targets of the EGFR signalling network, namely pERK and pAKT, were centrally analyzed together with the tumor suppressor p53 in erlotinib-treated pancreatic cancer patients: pERK overexpression showed a negative correlation with OS. Preliminary results in 50 patients analysed for p53 expression suggested an improvement in PFS and a higher rate of skin rash in patients whose tumors had a regular p53 expression compared to patients with a complete loss of p53. Our findings thus suggest that both pERK and p53 may serve as prognostic and/or predictive biomarkers in erlotinib-treated advanced pancreatic cancer.

## Electronic supplementary material

Additional file 1: Table S1: List of ethical committees List of German ethical committees that approved the AIO-PK0104 study. (DOC 36 KB)

Additional file 2: Figure S1: Immunohistochemistry staining for pERK in tissue specimens from AIO-PK0104. **A** Moderate pERK staining in pancreatic adenocarcinoma cells (score 4); **B** Strong pERK staining in pancreatic adenocarcinoma cells (score 9); (magnification x 200, for all figures). (ZIP 5 MB)

Additional file 3: Figure S2: Immunohistochemistry staining for p53 and pAKT in tissue specimens from AIO-PK0104. **A** No p53 staining in pancreatic adenocarcinoma cells with positive internal control (inflammatory cells, arrows), considered as complete loss of p53; **B** varying expression of p53 in tumor cells, considered as regular p53 expression; **C** strong homogenous p53 expression, considered as p53 overexpression (corresponding to mutation in p53); **D** weak nuclear pAKT expression; **E** strong nuclear pAKT reaction in tumor cells; **F** strong nuclear pAKT expression with additional cytoplasmic staining; (magnification x 200, for all figures). (TIFF 6 MB)
