# Supplementary material for: pERK, pAKT and p53 as tissue biomarkers in erlotinib-treated patients with advanced pancreatic cancer: a translational subgroup analysis from AIO-PK0104
Source: BMC Cancer. 2014 Aug 28;14:624. doi: 10.1186/1471-2407-14-624 (PMC4152581; doi:10.1186/1471-2407-14-624)
Supplement: Supplementary file 1 — Additional file 1: Table S1: List of ethical committees List of German ethical committees that approved the AIO-PK0104 study. (DOC 36 KB) [file 12885_2014_4797_MOESM1_ESM.doc]

**Table 1S**

List of German ethical committees that approved the AIO-PK0104 study:

- Ludwig-Maximilians-University of Munich

- Landesärztekammer Bayern

- University of Freiburg

- Landesärztekammer Baden-Württemberg

- Landesärztekammer Hessen

- Ärztekammer Sachsen-Anhalt

- Ärztekammer Nordrhein

- Ärztekammer Westfalen-Lippe

- Ärztekammer Niedersachsen

- Landesärztekammer Brandenburg

- Ärztekammer Schleswig-Holstein

- University of Frankfurt

- Ärztekammer Mecklenburg-Vorpommern

- Landesärztekammer Rheinland-Pfalz

- University of Cologne

- University of Göttingen

- Ärztekammer Hamburg

- University of Hannover

- University of Greifswald

- University of Essen

- University of Jena
