# Supplementary figures and images for: pERK, pAKT and p53 as tissue biomarkers in erlotinib-treated patients with advanced pancreatic cancer: a translational subgroup analysis from AIO-PK0104
Source: BMC Cancer. 2014 Aug 28;14:624. doi: 10.1186/1471-2407-14-624 (PMC4152581; doi:10.1186/1471-2407-14-624)

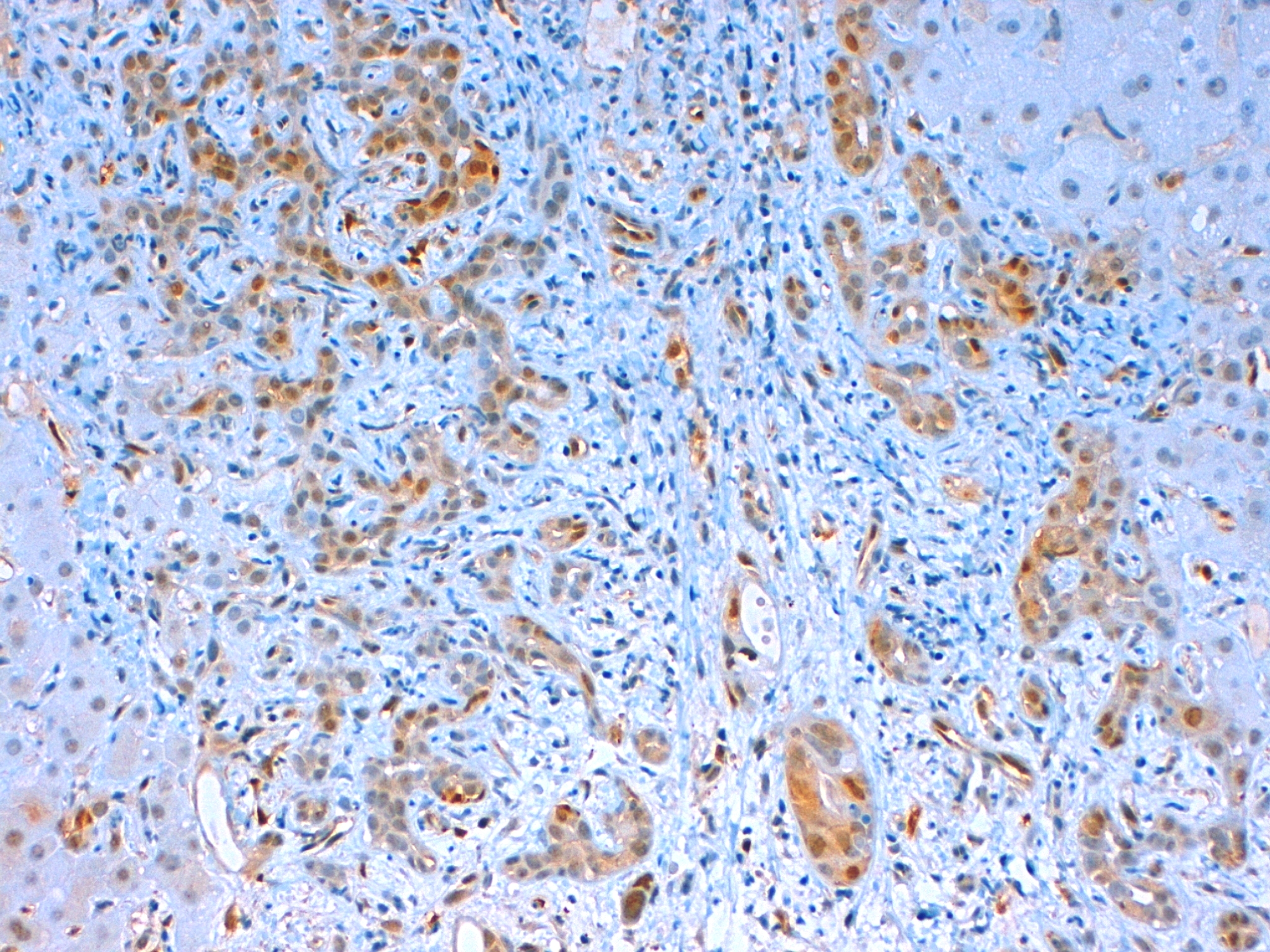

Supplement: Supplementary file 2 — Additional file 2: Figure S1: Immunohistochemistry staining for pERK in tissue specimens from AIO-PK0104. A Moderate pERK staining in pancreatic adenocarcinoma cells (score 4); B Strong pERK staining in pancreatic adenocarcinoma cells (score 9); (magnification x 200, for all figures). (ZIP 5 MB) [file 12885_2014_4797_MOESM2_ESM.zip › new/1733953403121345_fig3.jpeg]

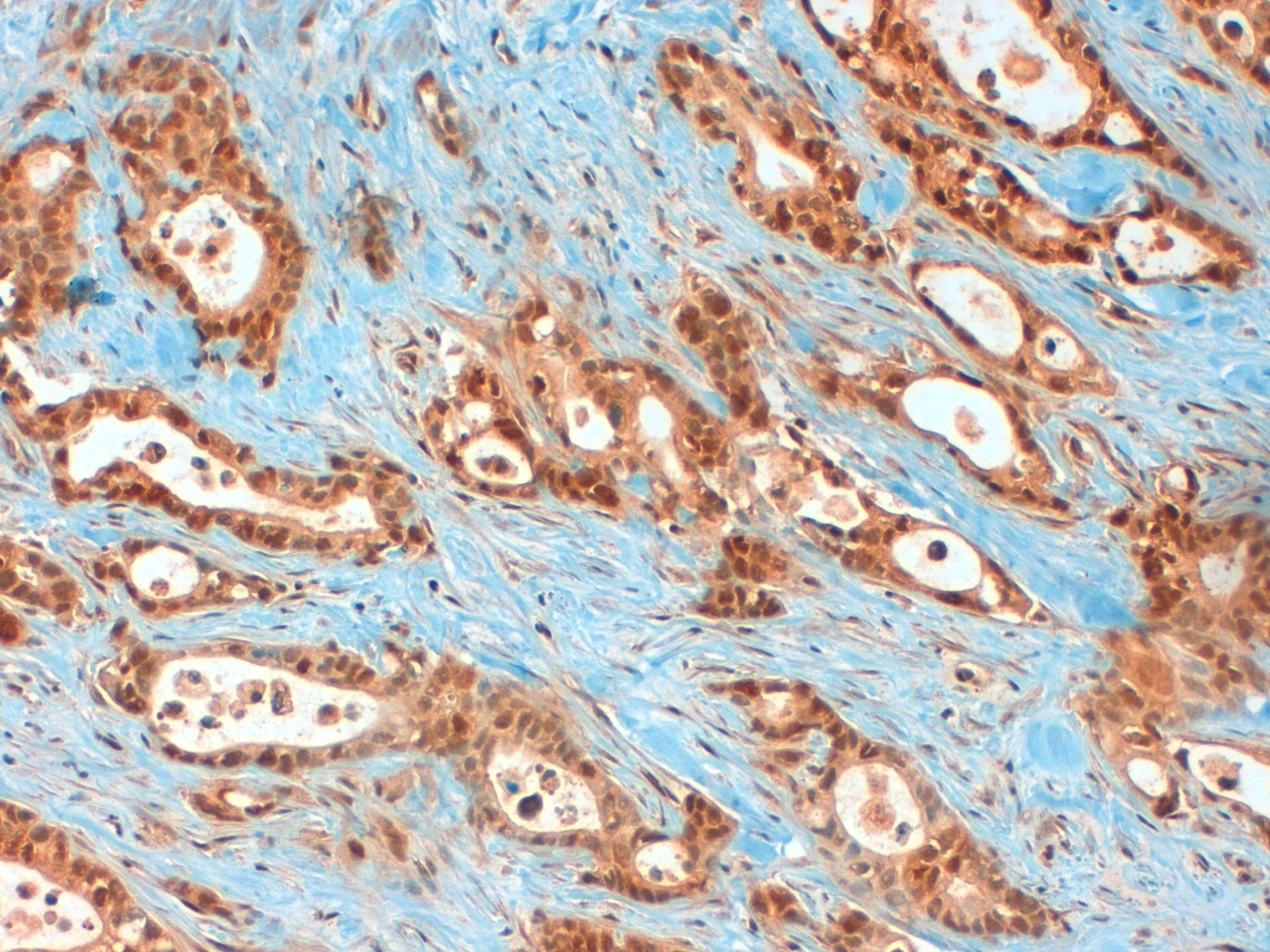

Supplement: Supplementary file 2 — Additional file 2: Figure S1: Immunohistochemistry staining for pERK in tissue specimens from AIO-PK0104. A Moderate pERK staining in pancreatic adenocarcinoma cells (score 4); B Strong pERK staining in pancreatic adenocarcinoma cells (score 9); (magnification x 200, for all figures). (ZIP 5 MB) [file 12885_2014_4797_MOESM2_ESM.zip › new/1733953403121345_fig4.jpeg]

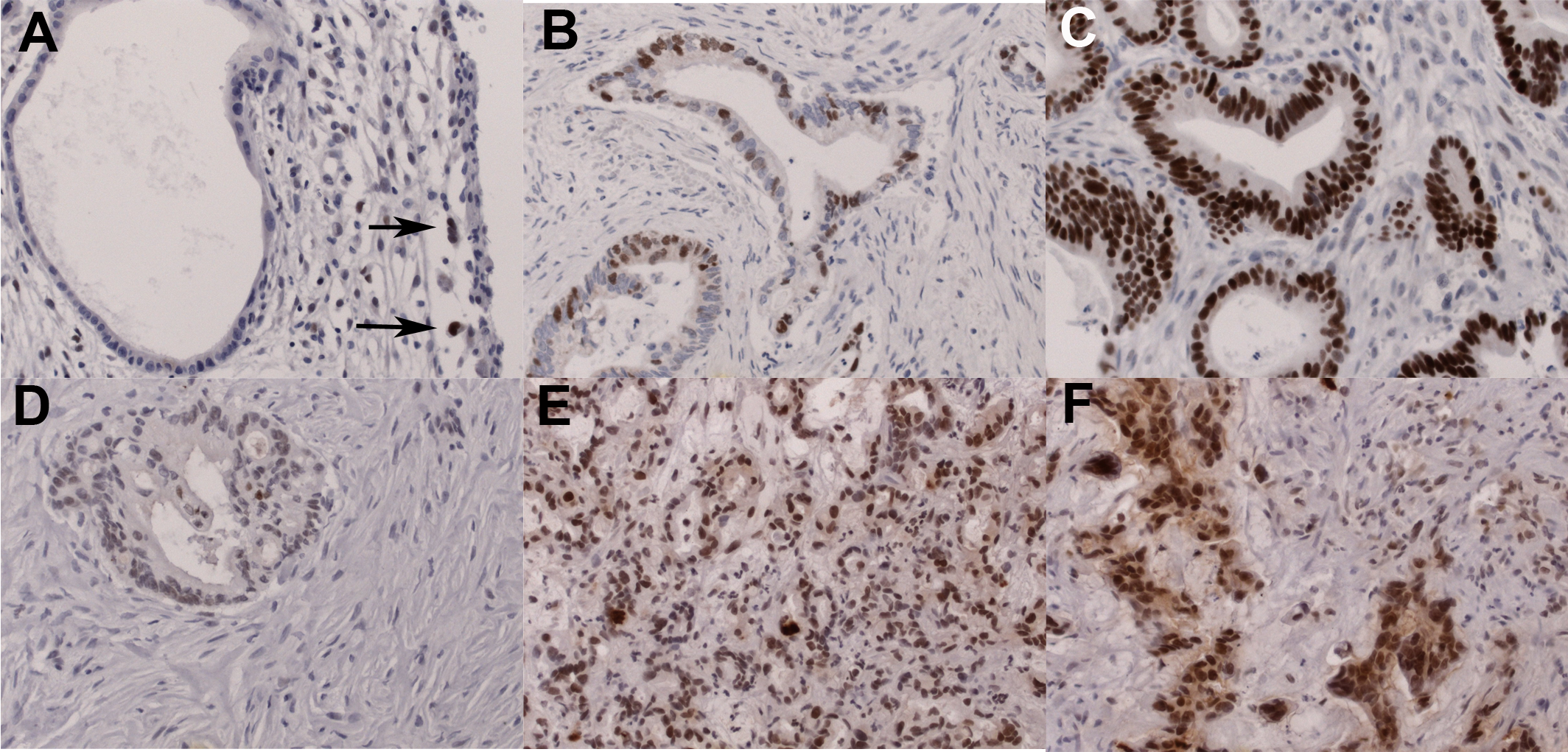

Supplement: Supplementary file 3 — Additional file 3: Figure S2: Immunohistochemistry staining for p53 and pAKT in tissue specimens from AIO-PK0104. A No p53 staining in pancreatic adenocarcinoma cells with positive internal control (inflammatory cells, arrows), considered as complete loss of p53; B varying expression of p53 in tumor cells, considered as regular p53 expression; C strong homogenous p53 expression, considered as p53 overexpression (corresponding to mutation in p53); D weak nuclear pAKT expression; E strong nuclear pAKT reaction in tumor cells; F strong nuclear pAKT expression with additional cytoplasmic staining; (magnification x 200, for all figures). (TIFF 6 MB) [file 12885_2014_4797_MOESM3_ESM.tiff]
